# Biosensing Platform for the Detection of Biomarkers for ALI/ARDS in Bronchoalveolar Lavage Fluid of LPS Mice Model

**DOI:** 10.3390/bios13070676

**Published:** 2023-06-25

**Authors:** Nuha Khalid Alekhmimi, Dana Cialla-May, Qasem Ramadan, Shimaa Eissa, Jürgen Popp, Khaled Al-Kattan, Mohammed Zourob

**Affiliations:** 1 Department of Chemistry, Alfaisal University, Al Zahrawi Street, Al Maather, AlTakhassusi Rd, Riyadh 11533, Saudi Arabia; 2 Leibniz Institute of Photonic Technology, Albert-Einstein-Straße 9, 07745 Jena, Germany; 3 Institute of Physical Chemistry (IPC) and Abbe Center of Photonics (ACP), Friedrich Schiller University Jena, Helmholtzweg 4, 07743 Jena, Germany; 4 Department of Chemistry, Khalifa University of Science and Technology, Abu Dhabi P.O. Box 127788, United Arab Emirates; 5 Advanced Materials Chemistry Center (AMCC), Khalifa University of Science and Technology, Abu Dhabi P.O. Box 127788, United Arab Emirates; 6 College of Medicine, Alfaisal University, Al Zahrawi Street, Al Maather, Al Takhassusi Rd, Riyadh 11533, Saudi Arabia

**Keywords:** ALI/ARDS, biosensors, PR3, human NE, MMP-2

## Abstract

Acute respiratory distress syndrome (ARDS) is a worldwide health concern. The pathophysiological features of ALI/ARDS include a pulmonary immunological response. The development of a rapid and low-cost biosensing platform for the detection of ARDS is urgently needed. In this study, we report the development of a paper-based multiplexed sensing platform to detect human NE, PR3 and MMP-2 proteases. Through monitoring the three proteases in infected mice after the intra-nasal administration of LPS, we showed that these proteases played an essential role in ALI/ARDS. The paper-based sensor utilized a colorimetric detection approach based on the cleavage of peptide–magnetic nanoparticle conjugates, which led to a change in the gold nanoparticle-modified paper sensor. The multiplexing of human NE, PR3 and MMP-2 proteases was tested and compared after 30 min, 2 h, 4 h and 24 h of LPS administration. The multiplexing platform of the three analytes led to relatively marked peptide cleavage occurring only after 30 min and 24 h. The results demonstrated that MMP-2, PR3 and human NE can provide a promising biosensing platform for ALI/ARDS in infected mice at different stages. MMP-2 was detected at all stages (30 min–24 h); however, the detection of human NE and PR3 can be useful for early- (30 min) and late-stage (24 h) detection of ALI/ARDS. Further studies are necessary to apply these potential diagnostic biosensing platforms to detect ARDS in patients.

## 1. Introduction

Acute respiratory distress syndrome (ARDS) is a life-threatening lung injury due to positive end-expiratory pressure resulting from the inflammatory response of the innate immune system. Acute lung injury (ALI) is a mild form of ARDS, with a ratio of arterial oxygen to inspired oxygen (PaO_2_/FIO_2_) of less than 300 mm Hg. This definition, which was updated in 2012 by the ARDS Definition Task Force, an international panel of experts, is known as the Berlin Definition. The Berlin Definition proposed three distinct classifications of ARDS based on the degree of hypoxemia: mild (200 mm Hg < PaO_2_/FiO_2_ ≤ 300 mm Hg), moderate (100 mm Hg < PaO_2_/FiO_2_ ≤ 200 mm Hg) and severe (PaO_2_/FiO_2_ ≤ 100 mm Hg) [1]. Despite removing the term “ALI” from the Berlin Definition, it is still used in research and clinical arenas [2].

ALI is considered an innate immune inflammatory response that can develop into a major clinical syndrome, ARDS, as well as interstitial edema and impaired gas exchange. Pulmonary neutrophil infiltration is a common pathophysiological characteristic of ALI/ARDS. Polymorphonuclear leukocytes (PMN) and pulmonary dysfunction are integral components of the alveolar infiltrate, which are mainly attributed to collateral damage induced by PMN invasion [3]. Lung injury and the infection-induced upregulation of proinflammatory cytokines (e.g., TNF-α and IL-8) and chemokines are remarkable manifestations of alveolar epithelial cells and the macrophage immune response [3]. This leads to neutrophil infiltration due to acute inflammatory disorders caused by proteases. Recent studies have shown that the neutrophil secretion of granule proteins leads to targeting and cleaving bacterial virulence factors [4]. It was found that neutrophil serine proteinases cleave bacteria in both humans and mice [5]. Proteinase 3 (PR3) can convert TNF-α cytokines and proceed to degrade membrane-bound pro-TNF-α cytokines, either by membrane-bound matrix metalloproteinase or a TNF-α converting enzyme, during the inflammation process [6].

ALI/ARDS is associated with high morbidity and mortality rates, with a wide variation in the reported population incidence, making it a global health problem. Despite improvements in treatment regimens in intensive care units, ARDS mortality has remained at around 40% for the last several years [7,8]. Therefore, the early diagnosis of ALI/ARDS cases is essential.

When a lung injury occurs, blood-circulating neutrophils migrate to the injured site and attach to the endothelium, releasing oxygen radicals and their internal constituents, which are stored in different granules [9]. The azurophil granules, for instance, consist of serine proteases, neutrophil elastase (NE), cathepsin G and PR3. Their main function is to release their granular content into the extracellular space via exocytosis [10], and break down the pathogens in phagosomes [6]. It is well known that the uncontrolled production of the serine protease, which PMNs produce, is associated with various inflammatory disease states, lung injuries and sepsis.

The PR3 protease is one of the main serine proteases that is secreted from neutrophils. It upregulates inflammation cascades [11,12] and induces PR3 lung damage. One of PR3’s functions is to initiate neutrophil apoptosis, which has various implications in autoimmune disease [13]. In early studies, PR3 was considered an enzyme that degrades elastin and causes pulmonary emphysema after intra-tracheal instillation in hamsters [14]. PR3 is also known to assign the same conserved catalytic cleft with 35% homology to cathepsin G [15] and 39% to neutrophil serine proteases 4 [16]. Moreover, neutrophil’s serine protease granule has a dynamic range that indicates PR3 when it rises to 13.4 mM [17]. Boxio et al. have shown that PR3 is a potent enzyme that can degrade E-cad, characterized as one of the most important, ubiquitous adherent junction proteins, and E-cad, a glycoprotein member of the cadherin superfamily that only overlaps the membrane once [18].

Matrix metalloproteinases (MMPs) are zinc-dependent proteinases capable of cleaving extracellular matrix proteins and enzymes, which have an important role in lung dysfunction [19,20,21,22]. MMPs modify cell–matrix and cell–cell interactions by their ability to cleave proteins such as collagen from large components to small proinflammatory mediators [23]. The activation of MMP-2 and MMP-9 by LPS induces the release of TNF-α, which can raise MMP-2 and MMP-9 levels in the blood of patients with sepsis in response to a Gram-negative bacterial infection. MMP-2/MMP-9 has been correlated with the severity of sepsis [24].

Various methods, such as CT scans [25,26,27,28] chest X-rays and MRI, are currently used for the diagnosis of ALI/ARDS [14,15]. However, using these methods requires well-trained laboratory personnel and costly instruments [28], and may increase lung injury risk [29,30,31]. The ALI “sniffer,” which can identify ALI with a sensitivity of 96% (95% CI 94–98) and a specificity of 89% (95% CI 88–90), can accurately determine if critically ill patients will develop ALI syndrome [32]. However, it is costly, and is only available in central healthcare centers and laboratories. The enzyme-linked immunosorbent assay (ELISA) and other immunoassays have also been employed to detect ALI/ARDS biomarkers in serum, plasma and bronchoalveolar lavage (BAL) [33,34], yet the ELISA is a time-consuming multi-step assay that requires costly reagents and well-trained personnel in centralized labs [35,36]. Therefore, developing rapid, low-cost ALI/ARDS methods is paramount.

The use of biosensors is an excellent alternative to traditional assays, in which the on-site monitoring of multiple biomarkers can be performed for the diagnosis of diseases. Few biosensors have been reported for the detection of PR3, MMP-2 and human NE (NE). A recent study by Griffith et al. identified the epitopes on a PR3-linked catalytic site using a resonant mirror biosensor for different patients with Wegener’s granulomatosis [19]. A computer-generated model showed that four out of five epitopes were quietly connected with the catalytic site and could form a contourable epitope. Wang et al. [25] developed an electrochemical peptide cleavage-based biosensor for MMP-2 detection with exonuclease III-assisted cycling signal amplification that could detect MMP-2 at concentrations as low as 0.15 pg/mL, with a dynamic range of 0.5 pg/mL–50 ng/mL. A recent study reported the detection of leukemia using electrochemical cytosensing to detect MMP-2 in human serum [26]. A colorimetric biosensor for the detection of NE has been reported for the diagnosis of periodontitis [27]. The absence of rapid, low-cost biosensors for detecting lung injuries, identifying and monitoring therapy, and predicting the prognosis of ARDS necessitates the availability of such diagnostic tools to detect ALI at an early stage before it develops into ARDS.

To the best of our knowledge, the diagnosis of ALI/ARDS through the detection of PR3, MMP-2 and NE as biomarkers has not been reported previously. In this study, we developed and characterized a paper-based multiplexing sensing method for detecting ALI/ARDS. Colorimetric paper-based biosensors are a simple detection method at ALI’s early and late stages. While paper has been used as a substrate for chemical analyses for many years, the concept of paper-based magnetic nanoparticles has only recently been introduced [36,37]. The easy synthesis of gold nanoparticles has led to increasing interest in applying AuNPs in interfacing biological recognition in several biosensor applications [38].

Specific peptide sequences for PR3, MMP-2 and NEs were conjugated with magnetic nanobeads, and a colorimetric probe was used to detect the cleavage of the peptides for the detection of ALI/ARDS in infected mice. The early detection of ALI/ARDS may enable an understanding of the mechanisms underlying pulmonary therapy for patients with ALI/ARDS, thus reducing the risk of life-threatening lung injuries.

## 2. Materials and Methods

### 2.1. Materials

The Gainland Chemical Company (Sandycroft, Deeside, UK) provided a Diff-Quick stain. The TNF-α and MIP-2 [39,40,41,42,43] kits were purchased from R&D System (Minneapolis, MN, USA). All of the reagents and solutions were prepared using deionized water and stored at −20 °C until their usage.

The MMP-2, PR3, NE peptides and lipopolysaccharide (LPS) *E. coli* 026:B6 [44,45,46,47,48,49,50,51,52,53,54] were synthesized by Pepmic Co. (Suzhou, Jiangsu, China). The PR3 peptide used was GIATDCMLMPEQ [55], the MMP-2 peptide was GPQVLANIPHFP [56], and NE peptide was APPEEIMDRQ [57]. Sodium phosphate and monopotassium phosphate, tris-base, sodium acetate, lipopolysaccharide (LPS) *E. coli* 026:B6, bovine serum albumin (BSA), sodium chloride, phosphate-buffered saline (PBS), ethyl-3-(3-dimethylaminopropyl)-carbodiimide (EDC), N-hydroxysuccinimide (NHS) and phosphate-buffered ethylenediaminetetraacetic acid (EDTA) were purchased from Sigma-Aldrich (Darmstadt, Germany).

### 2.2. Experimental Methods

#### 2.2.1. Mice Preparation

Wild-type C57BL/6J mice were purchased from Jackson Laboratories (Bar Harbor, ME, USA) at 6–10 weeks of age and 16–22 gms in weight. The mice were housed in the Department of Comparative Medicine at the King Faisal Specialist Hospital and Research Center (KFSH&RC) under specific pathogen-free conditions with a 12-h light/dark cycle and free access to food and water. The mice were used for in vivo experiments and as a source for collecting BAL fluid. The experimental protocols were revised and approved by the KFSH&RC Animal Care and Use Committee (RAC# 1MED 1672).

#### 2.2.2. LPS Model for ALI/ARDS

LPS from *E. coli* 026: B6 was used as an inhalation model for ALI/ARDS [41,42,43]. The LPS (50 μg in 50 μL normal saline) was administered intra-nasally by applying drops from a pipette placed on the nares. Each drop was allowed to be inhaled before the next drop was administered 1–2 min later. As a control, mice were given 50 μL of normal saline intra-nasally. The animal study was conducted according to a protocol approved by the KFSH&RC Animal Care and Use Committee (RAC# 1MED 1672).

#### 2.2.3. Bronchoalveolar Lavage Fluid (BALF)

After 30 min, 2 h, 4 h and 24 h of LPS treatment, the mice were anesthetized by the intraperitoneal injection of a mixer of ketamine (80 mg/kg) and xylazine (5 mg/kg). An incision was made in the neck, and the trachea was punctured with a 1 cc needle. Subsequently, the trachea was cannulated with a 24-gauge catheter and secured with 40 sutures. The lungs were lavaged with 6–8 aliquots of 0.6 mL of sterile PBS containing 0.1% EDTA. The BAL mice samples were stored at −80 °C until they were used again.

#### 2.2.4. Cytospin and Cell Count Differentials

The BAL fluid collected from the untreated and LPS-treated mice was centrifuged at 300× *g* for 10 min at 4 °C. The pellet cells were re-suspended in 5.0 mL of RPMI fresh media containing 10% fetal bovine serum and 5% penicillin/streptomycin. Subsequently, 200 μL was added to a glass slide and centrifuged using cytospin at 800 rpm for 3 min to allow the cells to adhere. The slides were left to dry overnight and stained with a Diff-Quick stain kit according to the manufacturer’s instructions. After drying, the slides were briefly placed in an eosin solution for 30 s, and in a methylene blue solution for 30 s. Then, they were washed with tap water to remove the residual dye. Next, the coverslips were placed on the slides. The AMΦ and neutrophils were identified morphologically and counted by two blinded observers (data not shown).

#### 2.2.5. Cytokines and Chemokine Measurements with ELISA

TNF-α and MIP-2 ELISA kits were used to assess AMΦ secretions according to the manufacturer’s instructions. BAL fluid was collected from the mice 4 h after LPS infection. ELISA kits were used to determine the anti-mouse TNF-α and MIP-2. After adding the substrate streptavidin and the termination of a 30-min reaction, the product was assessed calorimetrically with an ELISA reader. The results were compared to the TNF-α and MIP-2 standard curves described in the kits’ manuals.

#### 2.2.6. Conjugation of PR3, MMP-2 and Human NE Peptides with Magnetic Beads

A total of 100 µL of 1 mg/mL aliquots of peptide substrates were dissolved in dimethyl sulfoxide, then mixed with pre-washed magnetic beads and a freshly prepared EDC/NHS coupling solution. The mixture was rotated at room temperature and incubated overnight at 4 °C. The uncoupled peptides were removed by washing the beads 2–3 times with a washing buffer. The bead-activated peptides of PR3, MMP-2 and human NE were stored at 4 °C until further use.

#### 2.2.7. Sensing Platform Fabrication

**Scheme 1:**
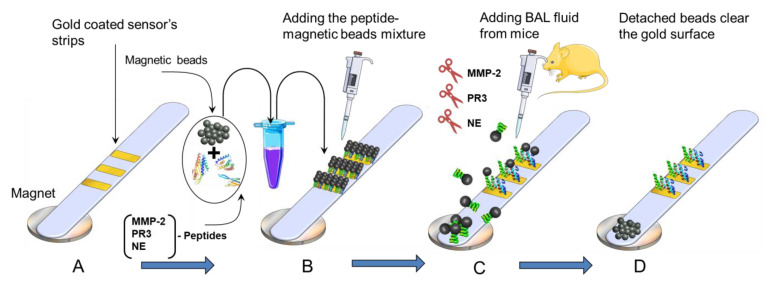
Schematic representation of the working principle of the paper-based ALI/ARDS sensor: (**A**) gold-coated adhesive tape was stacked over a paper substrate, (**B**) peptide-conjugated magnetic beads were added to the gold surface to form a functional sensor, (**C**) the LPS-treated BAL sample from the mice was added, and (**D**) uncovering the gold-colored surface due to protease peptide cleavage.

Self-adhesive sheets were purchased from Whatman (London, UK); they were coated with a gold layer with a thickness of 30 nm using a sputtering machine in a clean room at the King Abdullah University of Science and Technology (KAUST). The gold-coated sheet was cut into 1–2 mm sections and mounted over a plastic strip at a separation distance of 3 mm. The plastic strip was used as a physical support for the bio-functionalization process, as shown in Scheme 1A.

A layer of peptide–magnetic nanoparticle (MNPs) conjugates was immobilized onto the gold surface and allowed to dry at room temperature for 30 min (Scheme 1A). Subsequently, a permanent external magnet with dimensions of 10 mm × 10 mm × 5 mm and a magnetic flux density of 3300 Gauss at a 2 mm distance was passed over the functionalized strip to remove any unattached MNPs. Upon immobilization, the sensor’s gold surface was masked and turned black (Scheme 1B). After that, a round paper magnet was positioned 2–3 mm below the sensor strip. When the protease’s cleavage occurred, the black surface turned gold again due to the cleavage of the protease peptide that occurred when adding the sample containing the analytes, as shown in Scheme 1C.

#### 2.2.8. Quantitative Measurements of the Paper-Based Biosensing Platform

The images of the sensors were taken and saved in JPEG format, and processed using ImageJ software to calculate the quantitative data. The concentrations of the proteases of the paper-based biosensors and the multiplex data were calculated by dividing the cleaved area (yellow) by the total black sensor area. Quantitation experiments were conducted using different concentrations of proteases. The experiments were conducted in triplicate.

#### 2.2.9. In Vitro Testing Using FRET Assay (Fluorescence Resonance Energy Transfer Sensors)

The three FRET peptide substrates used in this study were purchased from Pepmic (Suzhou, China), with a purity greater than 95%. All of the peptides were dissolved in 100% dimethyl sulfoxide and stored at −80 °C. The proteolytic activities of the different substrates were individually monitored using 0.5 µL of 800 µM of FAM/DABCYL-modified peptides in 100 µL of MMP-2, PR3 and NE protease solutions in PBS buffer. BAL mice samples treated with normal saline were used.

The change in the fluorescence intensity of the FAM/DABCYL-modified peptides was monitored every minute for 2 h at 37 °C, at excitation and emission wavelengths of 485 and 535 nm, respectively. The comparative fluorescence intensity for the various protease probes in the BAL mice samples was analyzed by plotting the mean fluorescence intensity against time for each peptide. Normal saline was used in the blanks.

#### 2.2.10. Statistical Analysis

A *t*-test was used to compare the two means. An ANOVA test (San Diego, CA, USA) was used for multiple comparisons, followed by a *t*-test with Bonferroni corrections in Graphpad (San Diego, CA, USA).

## 3. Results and Discussion

Pre-clinical and clinical studies have indicated that the inflammatory response to direct and indirect insults to the lung plays a pivotal role in the pathogenesis of ALI/ARDS [2]. The early detection of bacterial infection can prevent the spread of ALI/ARDS and its progression. In this research, we developed a multiplexed paper-based sensor for the simultaneous detection of MMP-2, PR3 and NE proteases in an LPS mice model (mimic the ALI/ARDS). using specific peptides for the three proteases. The peptides were immobilized individually onto AuNP-modified paper-based surfaces (Scheme 1).

### 3.1. Assessing ALI/ARDS by Evaluating TNF-α/MIP-2 in a BAL Secretion after LPS Stimulation in Mice

**Figure 1:**
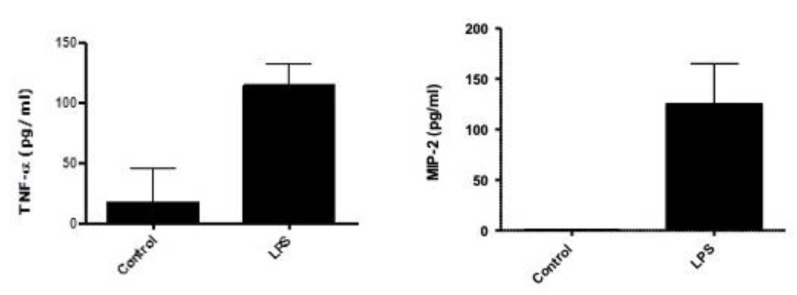
TNF-α and MIP-2 levels in BAL collected from post-LPS-treated mice (4 h) compared to normal saline (NS = control). TNF-α and MIP-2 significantly increased (10-fold) in BAL fluids, n = 3, *p* < 0.05.

The lung inflammation-associated upregulation of TNF-α and MIP-2 has been reported in several studies [38,39,40]. The LPS-intra-nasal injection of animal models has emphasized the role of TNF-α and MIP-2 secretion in ALI/ARDS [41,42]. An ELISA was used to measure the levels of LPS-induced prototypic cytokines, TNF-α and MIP-2. Figure 1 shows the comparison of control animals treated with normal saline (NS); TNF-α and MIP-2 secretions significantly increased (10-fold) in BAL fluids in response to LPS stimulation. The relative increments of the MIP-2 released were found to be higher than those of TNF-α in mice with ALI/ARDS, *p* < 0.05 (Figure 1). This finding, which agrees with those of previous studies [21,38,41,43], demonstrated that the TNF-α cytokine and MIP-2 chemokine play a significant role in upregulating lung inflammation in mice.

### 3.2. Quantitative Measurements for Various Proteases Using the Paper-Based Assay

**Figure 2:**
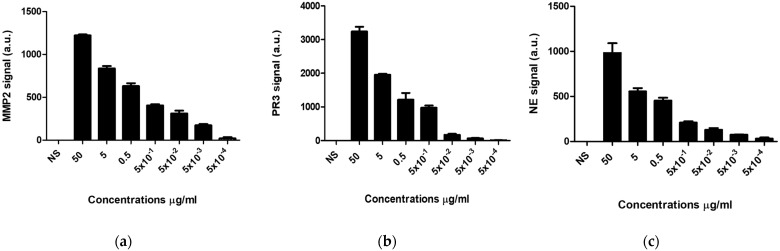
The response of protease sensors in normal saline (NS) and 50 µg/mL, 5 µg/mL, 0.5 µg/mL, 0.05 µg/mL, 50 ng/mL and 0.5 ng/mL for (**a**) MMP-2, (**b**) PR3 and (**c**) NE.

The sensors for the three proteases were prepared using the method reported previously [27,58,59]. Briefly, three specific peptides for PR3, MMP-2 and NE were attached covalently to magnetic nanobeads via the reaction of the peptide N terminal to the carboxyl-modified magnetic beads. The C-terminal of the peptide attached to the gold nanoparticle coated layer placed on a paper substrate via self-assembly. The attachment of the peptide–magnetic particle conjugates to the gold surface changes the color of the gold from yellow to black. On the other hand, the binding of the protease in the sample with the sensor surface leads to the cleavage of the peptide and the detachment of the magnetic particles from the sensor surface, which can then be removed using a magnetic placed underneath the paper. The sensors were tested to quantitatively detect the proteolytic activity of each protease (PR3, MMP-2 and NE). Upon proteolysis, the gold-colored sensor surface was revealed, leading to a significant change in the color, from black to yellow, which is visible to the naked eye. ImageJ software was used to quantitatively measure the current change upon peptide cleavage after binding with different concentrations of the three proteases. The peptide cleavage was correlated with the intensity of the revealed yellow color. Figure 2 shows that a gradual increase in the visible bare gold area was attained by increasing protease concentrations. It was observed that a high protease enzyme concentration allowed for faster peptide magnetic particles dissociation than low concentrations. As a negative control, normal saline showed no color change for the various proteases’ sensors (Figure 2).

The colorimetric biosensors enabled a limit of detection for as low as 0.5 ng/mL within one min, which was determined by identifying the lowest protease concentration capable of cleaving the covalently attached peptide black magnetic nanobeads, which revealed the sensors’ gold surface areas.

### 3.3. Measurements of Various Proteases in ALI/ARDS-Infected Mice

**Figure 3:**
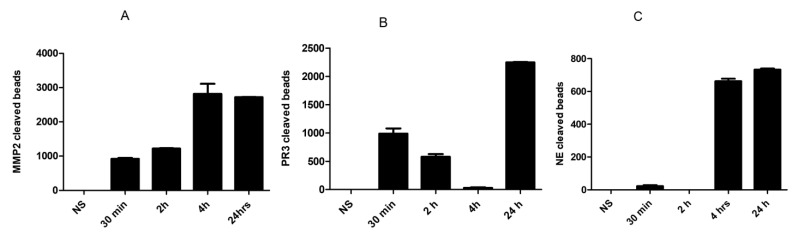
The profile of various proteases using the paper-based sensors after exposure to BAL collected from LPS-treated mice at different intervals. (**A**) MMP-2, (**B**) PR3 and (**C**) NE. Data were extracted using Image J software (n = 3).

Figure 3 shows the time-dependent concentration profiles of the secreted PR3, MMP-2 and NE in the BAL samples from infected mice taken at different time intervals (30 min, 2 h, 4 h and 24 h) after treatment with the LPS. Figure 3a shows that the concentration of the MMP-2 protease increased 30 min and 2 h after LPS administration, and significantly increased at 4 h and 24 h, implying that the MMP-2 peptide could serve as a biomarker for detecting early inflammation and late-stage ALI/ARDS. These results confirm those of previous studies [23,41,42,43,44,45,46,47,48,49,50,51,52,53]. There was an observable high cleavage of PR3 peptide with the addition of BAL mice samples treated with LPS for 30 min, 2 h and 24 h (Figure 3b). However, no peptide cleavage was observed after 4 h of LPS administration, which indicates that the intra-nasal administration of LPS resulted in a cell count differential increase in PMN in the BAL fluid (data not shown). Therefore, these data suggest that PR3 can be used for the early detection (30 min–2 h) of early-stage ALI/ARDS and severe ARDS (after 24 h). Figure 3b showed significant increases in PR3 with time, except for the drop shown after 4 h.

NE plays an essential role in severe acute lung injuries. Elastase enzymes are secreted in ALI/ARDS during the inflammation process. Figure 3c shows a clear increase in NE peptide when exposed to BAL collected from LPS-treated mice samples after 4 and 24 h. Slight cleavage was detected at 30 min, but no cleavage was detected at 2 h. These results established that the NE biomarker could be useful for detecting late-stage ALI/ARDS. It is worth mentioning that the specificity of the biosensor was tested using nonspecific proteases and no cross reactivity was detected, indicating the high specificity of this assay.

### 3.4. Comparison of the Paper-Based Sensors with Fluorescence Resonance Energy Transfer (FRET) Sensors

**Figure 4:**
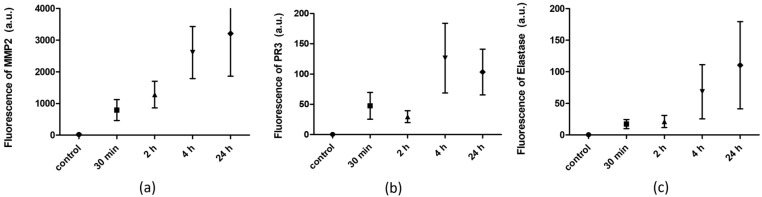
Comparison of FRET-based peptide substrate and paper-based biosensors with (**a**) MMP-2; (**b**) PR3; (**c**) NE (*p* value = 0.001, n = 4).

FRET sensors have recently been used to quantitatively detect protease activity [55]. The results of the peptide-conjugated magnetic nanobeads with paper-based sensors were compared with peptides containing fluorescence and a quencher in a solution. The peptides were labeled with FAM fluorophore on the N-terminal and quencher DABCYL on the C-terminal. The FRET-specific peptides for the various proteases were incubated with different sets of BAL samples collected at different times following the introduction of LPS. Figure 4 shows the FRET results for the proteases MMP-2, PR3 and NE upon exposure to LPS samples collected at different times (0, 30 min, 2 h, 4 h and 24 h). A significant increase in fluorescence intensity was observed at 30 min, 2 h, 4 h and 24 h following ALI/ARDS, but no increase was seen in the BAL sample treated with NS. These results were in agreement with those of MMP-2 paper-based sensing cleavage, indicating that the colorimetric paper-based sensor can be used as a rapid detection tool. Meanwhile, PR3 FRET peptide sequences only led to a slight increase in the intensity of the fluorescence at 30 min and 2 h, but a significant increase after 24 h when exposed to the LPS-treated animal BAL samples (Figure 4b). Figure 4c also indicates a significant increase in fluorescence after 4 and 24 h after LPS administration. These results agree with those obtained using paper-based sensors. Eventually, the signal intensity profiles for different peptides at various times could detect ARDS in the early, severe stages of lung injuries in mice.

## 4. Conclusions

This study used proteases released from BAL in LPS-treated mice as biomarkers for the diagnosis of ALI/ARDS. Paper-based sensors provided a simple yet sensitive and rapid method of detection that can be read visually without laboratory-based equipment. The cleavage of the peptide–AuNPs conjugate, which was related to the protease concentration, resulted in exposing the sensor surface and revealing a gold color from a surface that was initially covered with black beads. The sensor showed good sensitivity, with a limit of detection of 5 × 10^−4^ µg/mL and a high specificity, as demonstrated by the multiplexed detection of three different proteases on the same paper sensor. We found that MMP-2 was the best biomarker for ALI/ARDS, and was able to detect the disease at all stages; thus, this multiplexing platform could provide very useful information for patients with ALI/ARDS. The reported multiplexed paper-based assay can provide rapid diagnosis of lung inflammation, showing potential advantages over existing traditional methods in terms of its cost reductions, simplicity and fast response.

## Data Availability

Not applicable.
